# Incidence and prognostic significance of malignant arrhythmias during (repetitive) Holter electrocardiograms in patients with pulmonary hypertension

**DOI:** 10.3389/fcvm.2023.1084051

**Published:** 2023-04-17

**Authors:** Dirk Bandorski, Sebastian Heibel, Reinhard Höltgen, Harilaos Bogossian, Hossein Ardeschir Ghofrani, Markus Zarse, Henning Gall

**Affiliations:** ^1^ Faculty of Medicine, Semmelweis University, Budapest, Hungary; ^2^ Medical Clinic III, Sana Klinikum Offenbach, Offenbach am Main, Germany; ^3^ Klinikum Westmünsterland, St. Agnes-Hospital Bocholt-Rhede, Bocholt, Germany; ^4^ Evangelical Hospital Hagen-Haspe, Hagen, Germany; ^5^ The German Center for Lung Research (DZL), University of Giessen and Marburg Lung Center (UGMLC), Giessen, Germany; ^6^ Cardiology Department, School of Medicine, Witten/Herdecke University, Witten, Germany

**Keywords:** pulmonary hypertension, PH, Holter ECG, arrhythmias, prognosis

## Abstract

**Background:**

In patients with pulmonary hypertension (PH), increased pulmonary vascular resistance (PVR) may lead to increased right ventricular afterload and cardiac remodelling, potentially providing the substrate for ventricular arrhythmias. Studies dealing with long term monitoring of patients with PH are rare. The present study evaluated the incidence and the types of arrhythmias retrospectively recorded by Holter ECG in patients with newly detected PH during a long-term Holter ECG follow-up. Moreover, their impact on patient survival was evaluated.

**Patients and methods:**

Medical records were screened for demographic data, aetiology of PH, incidence of coronary heart disease, level of brain natriuretic peptide (BNP), results from Holter ECG monitoring, 6-minute walk test distance, echocardiographic data and hemodynamic data derived from right heart catheterization. Two subgroups were analyzed: 1. patients (*n* = 65) with PH (group 1 + 4) and derivation of at least 1 Holter ECG within 12 months from initial detection of PH and 2. patients (all PH etiologies, *n* = 59) with 3 follow-up Holter ECGs. The frequency and complexity of premature ventricular contractions (PVC) was classified into “lower” and “higher” (=non sustained ventricular tachycardia, nsVT) burden.

**Results:**

Holter ECG revealed sinus rhythm (SR) in most of the patients (*n* = 60). Incidence of atrial fibrillation (AFib) was low (*n* = 4). Patients with premature atrial contractions (PAC) tend to have a shorter period of survival (*p* = 0.098), PVC were not correlated with significant survival differences. During follow-up PAC and PVC were common in all PH groups. Holter ECG revealed non sustained ventricular tachycardia in 19/59 patients [(32.2%); *n* = 6 during first Holter-ECG, *n* = 13 during second/third Holter-ECG]. In all patients suffering from nsVT during follow-up previous Holter ECG revealed multiform/repetitive PVC. PVC burden was not linked to differences in systolic pulmonary arterial pressure, right atrial pressure, brain natriuretic peptide and results of six-minute walk test.

**Conclusion:**

Patients with PAC tend to have a shortened survival. None of the evaluated parameters (BNP, TAPSE, sPAP) was correlated with the development of arrhythmias. Patients with multiform/repetitive PVC seem to be at risk for ventricular arrhythmias.

## 1. Introduction

Pulmonary hypertension (PH) is defined as an elevation of the mean pulmonary arterial pressure (mPAP) > 20 mmHg at rest (1)—an earlier definition included mPAP < 25 mmHg (2, 3)—concomitant with either pulmonary vascular resistance (PVR) ≥ 3 Wood units (WU) and pulmonary capillary wedge pressure (PcWP) ≤ 15 mmHg in precapillary forms or PVR < 3 WU and PcWP > 15 mmHg in postcapillary forms (4). Increased PVR leads to increased afterload of the right ventricle and cardiac remodelling (5), which could provide the substrate for cardiac arrhythmias (6, 7). In most studies regarding cardiac rhythm in PH patients have undergone repeated standard electrocardiograms (ECG) to evaluate supraventricular arrhythmias (8–12). In three studies, the authors evaluated the incidence of ventricular arrhythmias in patients who underwent 24-h Holter ECG for close monitoring of their heart rhythm (13–15). There are few data about the onset of arrhythmias after the first diagnosis of PH and in none of the recent studies patients underwent repeated Holter ECG monitoring.

We performed the present study to evaluate the incidence and type of arrhythmias identified by Holter ECG in patients who have been newly diagnosed with PH and who underwent long-term Holter ECG follow-up. In addition, we examined the influence of the arrhythmias found on patient survival.

## 2. Methods

Data of the patients of the PH department of University Hospital of Giessen, Germany from a period of 10 consecutive years were retrospectively analysed. They included patients with PH (any subgroup) and a recording of at least one Holter ECG within 12 months from the initial detection of PH. Medical records were screened for the following data: demographic data, aetiology of PH, incidence of coronary artery disease (CAD), level of brain natriuretic peptide (BNP), results from Holter ECG monitoring, 6-minute walk test (6MWT) distance, echocardiographic data, and hemodynamic data derived from right heart catheterisation (RHC). The findings were obtained from patient files; thus, the number in the subgroups can deviate from the total number of patients if information is missing.

**Figure 1:**
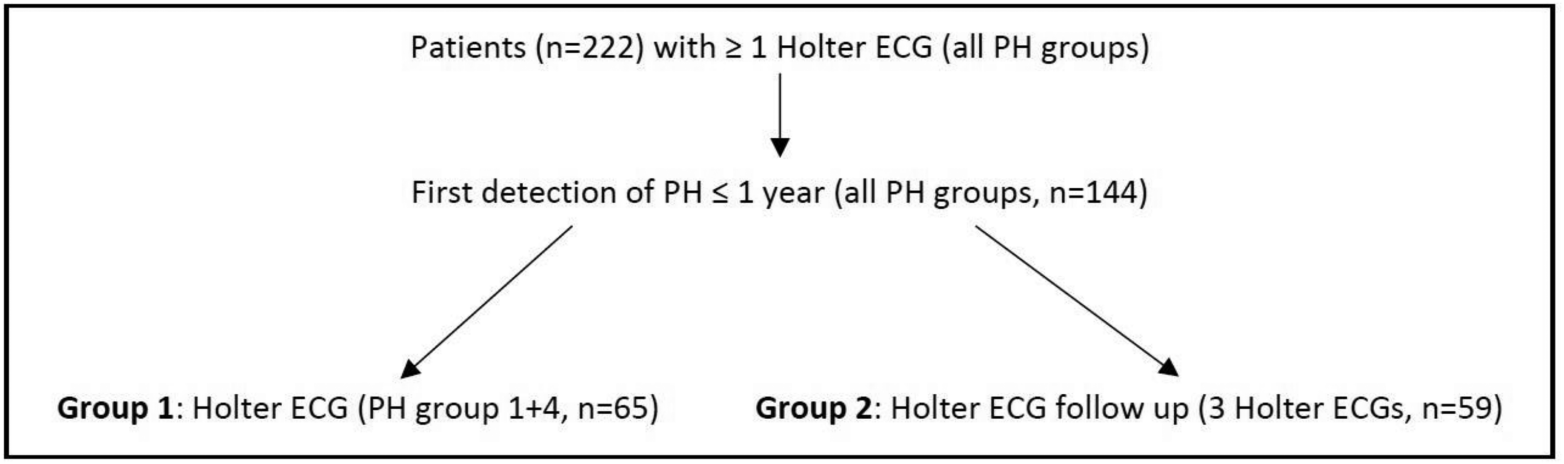
Description of the study population. ECG = electrocardiogram; PH = pulmonary hypertension.

These data are part of the doctoral thesis of Sebastian Heibel. To match the data with other studies (8, 10–12), a subgroup of patients with group 1 PH [pulmonary arterial hypertension (PAH)] and group 4 [chronic thromboembolic pulmonary hypertension (CTEPH)] was formed. To evaluate heart rhythm over the disease process, another subgroup was created, including all patients with three follow-up Holter ECGs (Figure 1) of all PH aetiologies. Patients were seen in a structured follow up (according to the clinic's internal procedure) every 3 to 6 months in the pulmonary hypertension outpatient department with clinical examination, echocardiography and Holter monitoring based on medical necessity. Data of last contact and corresponding survival status were noted.

The study and the study design were approved by the institutional review board (Ethics Commission of the Faculty of Medicine, Justus Liebig Universität Giessen, reference number: 107/13). The study was registered in ClinicalTrials.gov (ID: NCT05496504, Protocol ID: PH09_08_2022).

### 2.1. Holter electrocardiograms

Patients underwent Holter ECG monitoring (GETEMED, Teltow, Germany) for 24 h to detect arrhythmias and heart rate variability (HRV). Detected arrhythmias were classified following the terminology of the European Society of Cardiology (ESC) (16, 17). In accordance with a previous study from our group (14), the following arrhythmias were considered potentially prognostic relevant: infrahisian atrioventricular (AV) block (second-degree type 2 and third-degree AV block are associated with a poor outcome if undetected and/or untreated) (18), atrial flutter (due to its danger of 1:1 AV conduction) and ventricular tachycardias [increased rate of sudden cardiac death (SCD) in patients with a reduced ejection fraction] (17, 19).

Non-life-threatening arrhythmias were defined as follows: premature atrial contractions (PAC), non-sustained or sustained supraventricular tachycardias (e.g., atrial tachycardia or atrial fibrillation, which are not reported to be related to SCD) and isolated premature ventricular contractions (PVC). PVC were graded by the physicians analysing the Holter ECGs using the Lown classification as a surrogate parameter (20). The predictive value of Lown's grading system has only been proved for CAD or for arrhythmias after myocardial infarction (MI) and the risk of a prognostic deterioration does not generally increase with the class (21). Nevertheless, the occurrence of a high burden of PVCs as well as nsVT are seen as risk factors for SCD (22) and this classification has become established in clinical practice for long-term ECG diagnosis, including in patients with other underlying diseases—for example, PH (23). Lown grade 4b was classified as non-sustained ventricular tachycardia (nsVT, ≥3 beats in duration, terminating spontaneously in less than 30 s) (24). Since the use of the classification is no longer recommended, we differentiated the arrhythmic burden by PVCs between patients with lower burden (Lown 0–4a) and higher burden (Lown 4b).

The results of Holter ECG recordings were correlated with echocardiography, RHC data, laboratory values and the 6MWT distance.

### 2.2. Six-Minute walk test

In accordance with the guidelines of the American Thoracic Society (ATS), patients walked along a 100-foot floor at their own pace to cover as much distance as possible in 6 min (25). The total distance walked was determined.

### 2.3. Echocardiography

Transthoracic echocardiographs were evaluated by experienced investigators (GE, Boston, Massachusetts, United States). The following diameters and valves were measured: the left atrial diameter (edge-to-edge method, parasternal long axis view), the right atrial diameter (four-chamber view, measured at end systole), the left ventricular ejection fraction (LVEF, biplane Simpson method, two- and four-chamber view), tricuspid annular plane systolic excursion (TAPSE), and systolic pulmonary artery pressure (sPAP). All measurements were carried out in accordance with the guidelines of the American Society of Echocardiography (26–29).

### 2.4. Right heart catheterisation

RHC was performed *via* the right jugular vein. Hemodynamic measurements included mean right atrial pressure (mRAP), mPAP, PcWP, PVR, the cardiac index (CI, thermodilution method), and mixed venous oxygen saturation (SvO_2_).

### 2.5. Statistical analysis

After pseudonymisation, all statistical analysis was performed using SPSS® Statistics, Versions 21 and 28 (IBM Corp., Armonk, NY, United States). The patient data are presented as the mean [standard deviation (SD)] or median [interquartile range (IQR)]. Statistical evaluation included possible correlations between baseline and follow-up Holter ECG monitoring and echocardiography as well as clinical and laboratory parameters. Pearson's chi-squared test or Fisher's exact test was used for descriptive statistical comparisons. To control the type I error of multiple testing, one-way analysis of variance (ANOVA) followed by Tukey's honestly significant difference (HSD) *post hoc* test was used for multiple comparisons. Levene's test for homogeneity of variances was performed in every analysis. Mortality analysis was carried out using the Kaplan–Meier (KM) method to analyse “time-to-event” data. The event status consisted of two mutually exclusive events: censored (loss to follow-up) or event (death). The Mantel–Cox log rank test was used to test the equality of the overall survival distributions between the groups. For all analyses, *p* < 0.05 was considered a statistically significant result. *p*-values between 0.05–0.1 were considered as trend towards statistical significance.

## 3. Results

### 3.1. Group 1 (PH group 1, 1′, and 4, baseline Holter ECG)

Sixty-five patients (female, *n* = 43; male, *n* = 22) were enrolled. The mean age was 64 ± 15 years. 21 patients (32%) had PAH (group 1), 3 patients (5%) had group 1′ PH and 41 patients (63%) had CTEPH (group 4). 15 patients (23%) had an anamnesis of CAD (group 1, *n* = 5; group 4, *n* = 10) without the need for treatment or myocardial infarction. The data of echocardiography, right heart catheterization, BNP and 6MWT are shown in Table 1.

**Table 1:** Group 1 baseline values.

PH group	CAD	Echocardiography	Right Heart Catheterisation	BNP	6MWT
1, 1′, 4	PH group 1: *n* = 5	**TAPSE:** 17 ± 5 mm	**RAP:** 7.5 ± 5.6 mmHg	296 ± 433 pg/ml (age- and sex-matched normal value: <36–176 pg/ml)	326 ± 142 m
		**LV-EF:** 64 ± 8%	**mPAP:** 50 ± 14 mmHg		
		**sPAP:** 73 ± 25 mmHg	**PVR:** 915 ± 498 dyn × s × cm^−5^ (11.4 ± 6.2 WU)		
	PH group 4: *n* = 10	**LA:** 41 ± 8 mm	**PcWP:** 8.8 ± 4.4 mmHg		
		**RA:** length 51 ± 9 mm, width 49 ± 9 mm	**SvO_2_:** 60% ± 9%		
			**CI:** 2.2 ± 0.6 L/min/m²		

PH, pulmonary hypertension; CAD, coronary artery disease; TAPSE, tricuspid annular plane systolic excursion; LV-EF, left ventricular ejection fraction; sPAP: systolic pulmonary arterial pressure; LA, left atrium; RA, right atrium; RAP, right atrial pressure; mPAP, mean pulmonary arterial pressure; PVR, pulmonary vascular resistance; PCWP, pulmonary capillary wedge pressure; SvO_2_, venous oxygen saturation; CI, cardiac output (liters (l) per minute (min) per square meters (m^2^); BNP, brain natriuretic peptide; 6MWT, Six-Minute Walk Test.

#### 3.1.1. Holter electrocardiogram monitoring

Holter ECG revealed sinus rhythm (SR) in 60 patients (92%; CAD, *n* = 15) and atrial fibrillation in 4 patients (7%; CAD: *n* = 1). Mean heart rate was 76 ± 11 beats per minute (bpm), minimal heart rate 60 ± 11 bpm, and maximal heart rate 118 ± 26 bpm. The standard deviation of NN intervals as an indicator of HRV (SDNN) was decreased, with a mean of 77 ± 11 ms (normal value:141 ± 39 ms). PAC occurred in 19 patients (PH group 1: *n* = 6, CAD: *n* = 3, PH group 4: *n* = 13, CAD: *n* = 3). PVC were found in 51 patients (PH group 1: *n* = 17, CAD: *n* = 5; PH group 4: *n* = 34, CAD = 10). The most complicated PVC were salves of ≥3 PVC (nsVT, *n* = 7).

Due to low number of patients, no subgroup analysis was performed for the above-mentioned parameters.

#### 3.1.2. Heart rhythm and survival

No significant survival difference between groups with SR and with AFib was found taking into account the small sample size of the AFib group (*n* = 4).

#### 3.1.3. Premature atrial contractions, premature ventricular contractions and survival

**Figure 2:**
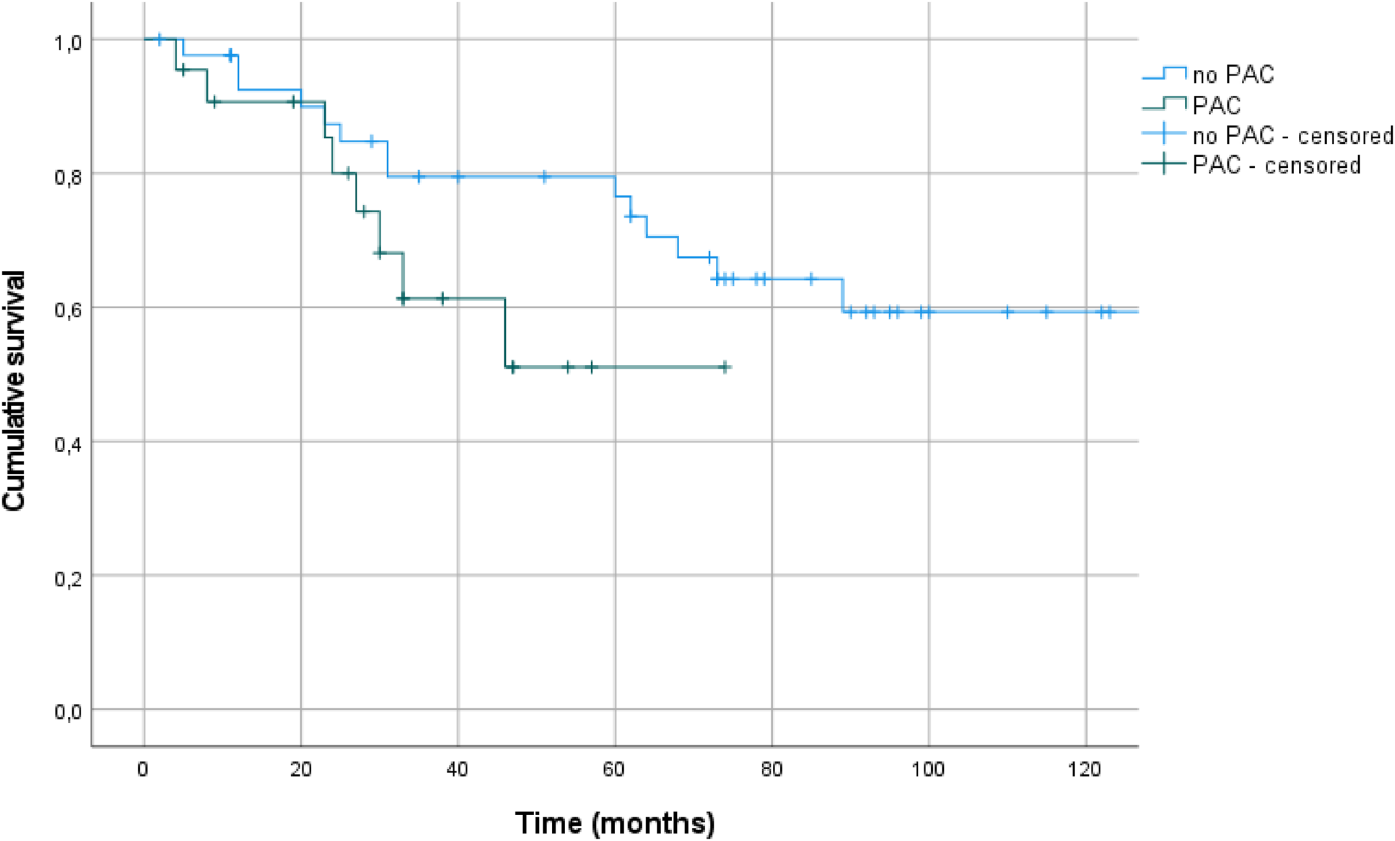
Kaplan–Meier plots (+ = censored data) for survival of patients with (*n* = 19) or without (*n* = 46) premature atrial contractions (PAC).

The 1-, 3-, and 5-year survival rates were 92.5%, 79.5%, and 76.5%, respectively, for patients without PAC vs. 90.7%, 61.3%, and 51.1%, respectively, for patients with PAC (Figure 2; log rank test *p* = 0.098).

**Figure 3:**
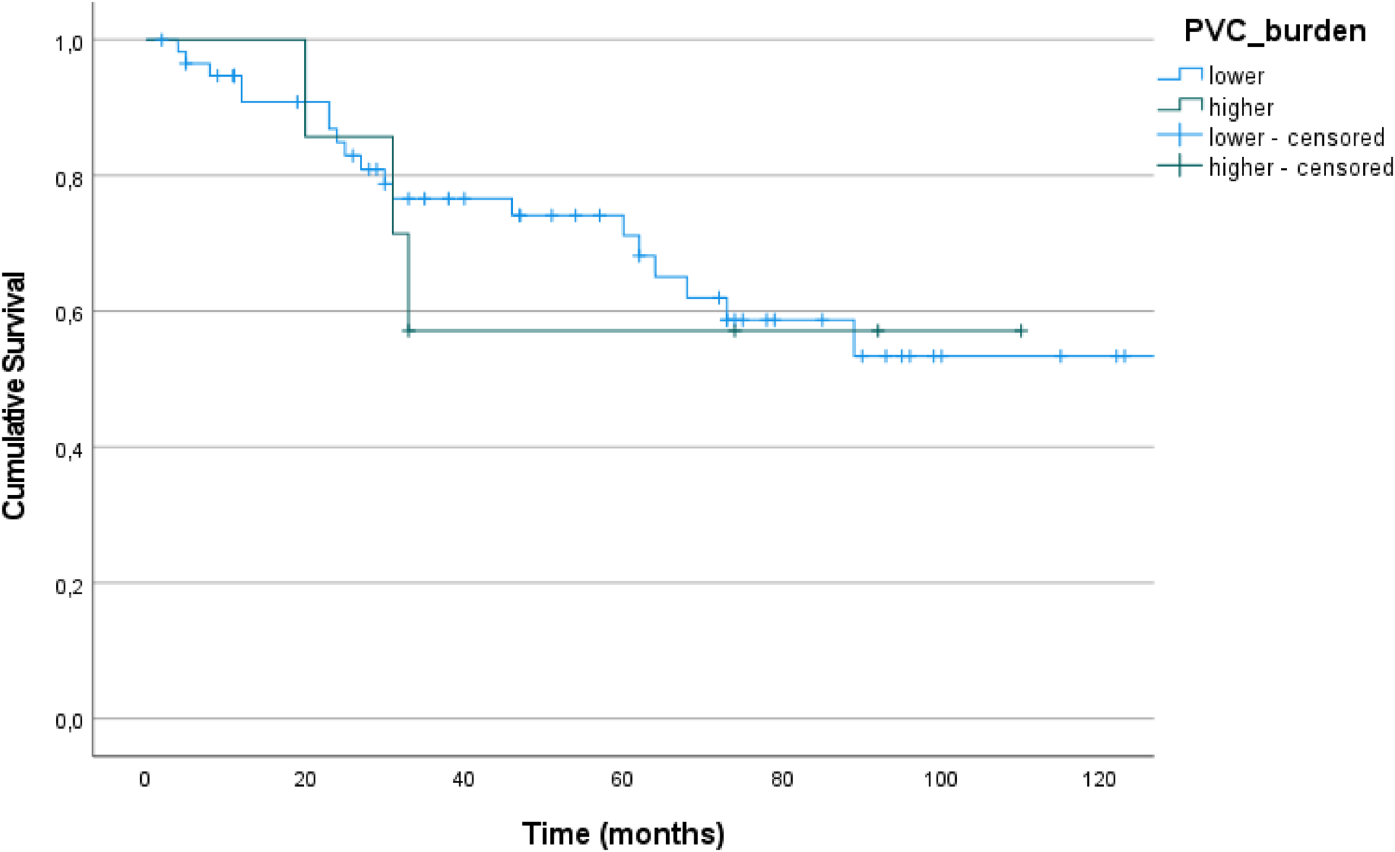
Kaplan–Meier plots (+ = censored data) for survival of patients with lower (*n* = 51) and higher (*n* = 7) PVC burden.

Survival data showed no differences between patients with lower and higher PVC burden (log rank test *p* = 0.849, Figure 3).

### 3.2. Group 2 (all PH groups, three follow-up Holter electrocardiograms)

Fifty nine patients (female, *n* = 29; male, *n* = 30) underwent three Holter ECGs. The median time between the first and the second examination was 3 months [IQR 1–10]. The distribution of time interval from Holter examination 1 to Holter examination 3 was left skewed. The median was 14 months [IQR 6–34]. The mean age at the first Holter monitoring was 65 ± 13 years. There were patients in all PH groups: group 1, *n* = 14; group 1′, *n* = 3; group 2, *n* = 9; group 3, *n* = 12; group 4, *n* = 19; and group 5, *n* = 2. Sixteen patients had an anamnesis of CAD (group 1, *n* = 5; group 2, *n* = 4; group 3, *n* = 3; group 4, *n* = 4; and group 5, *n* = 0) without MI.

#### 3.2.1. Holter electrocardiogram monitoring

Holter ECG revealed SR in most patients. AFib and other heart rhythms occurred less frequently (Table 2).

**Table 2:** Number of patients with each heart rhythm in the three consecutive Holter electrocardiograms.

	Rhythm			
	SR	AFib	PMR	FAT
Holter ECG 1	51 (13)	6 (2)	1 (1)	–
Holter ECG 2	47 (13)	9 (3)	2 (0)	–
Holter ECG 3	51 (14)	5 (1)	2 (1)	1 (0)

Numbers in brackets = number of patients with CAD. FAT, focal atrial tachycardia; AFib, atrial fibrillation; PMR, pacemaker rhythm; SR, sinus rhythm.

Holter ECG revealed a mean heart rate of 76 ± 14 bpm at Holter ECG 1, 77 ± 14 bpm at Holter ECG 2, and 79 ± 14 bpm at Holter ECG 3. The minimal heart rate was 58 ± 13, 59 ± 11, and 61 ± 12 bpm (Holter ECG 1/2/3) and the maximal heart rate 115 ± 23, 114 ± 18, and 119 ± 22 bpm (Holter ECG 1/2/3). The SDNN interval was 76 ± 13 ms, 71 ± 15 ms and 76 ± 14 ms for Holter ECG 1, 2 and 3, respectively.

PVC occurred frequently in all patients over the entire period. There was no apparent trend for PAC (Table 3). No significant differences between PH groups were found (Holter ECG 1, 2, and 3: PVC, *p* = 0.413/0.342/0.307, respectively; PAC, *p* = 0.40/0.696/0.904, respectively).

**Table 3:** Premature ventricular contractions (PVC) and premature atrial contractions (PAC) for each Holter electrocardiogram (ECG).

		PH group					
		1	1'	2	3	4	5
PVC Holter ECG 1	No	2	1	1	1	0	0
	Yes	12	2	8	11	19	2
PVC Holter ECG 2	No	0	0	1	0	0	0
	Yes	14	3	8	12	19	2
PVC Holter ECG 3	No	1	0	3	2	1	0
	Yes	13	3	6	10	18	2
PAC Holter ECG 1	No	8	2	7	7	13	0
	Yes	6	1	2	5	6	2
PAC Holter ECG 2	No	9	2	7	6	13	2
	Yes	5	1	2	6	6	0
PAC Holter ECG 3	No	9	2	7	7	14	1
	Yes	5	1	2	5	5	1

PH, pulmonary hypertension.

Because of a small number of cases, no analysis of the subgroups (heart rhythm) was carried out for the parameters mentioned.

#### 3.2.2. Premature ventricular contractions and echocardiography, brain natriuretic peptide, and six-minute walk test results during Holter electrocardiogram follow-up

All patients had a normal LVEF at the first echocardiography (lower/higher PVC burden: 60 vs. 59%; *p* = 0.340). There were no significant differences in echocardiography, BNP, and 6MWT between patients with lower or higher burden of PVC (Table 4).

**Table 4:** PVC burden and six-minute walk test (6MWT), echocardiographic data, and brain natriuretic peptide during Holter electrocardiogram (ECG) follow-up.

	PVC Burden	Holter ECG 1	Holter ECG 2	Holter ECG 3
**TAPSE** (*p* = 0.140 /*p* = 0.112/*p* = 0.125)	Lower	18	18	19
	Higher	23	22	16
**sPAP** (*p* = 0.317 /*p* = 0.628/*p* = 0.821)	Lower	66	61	61
	Higher	81	55	60
**RAP** (*p* = 0.810 /*p* = 0.851/*p* = 0.585)	Lower	6	6	5
	Higher	7	6	6
**BNP** (*p* = 0.568 /*p* = 0.707/*p* = 0.866)	Lower	233	272	322
	Higher	327	343	296
**6MWT** (*p* = 0.953 /*p* = 0.295/*p* = 0.678)	Lower	320	344	348
	Higher	316	409	330

BNP, brain natriuretic peptide; RAP, right atrial pressure; sPAP, systolic pulmonary arterial pressure; TAPSE, tricuspid annular plane systolic excursion; PVC, premature ventricular contractions. *p* values between lower/higher PVC burden at Holter ECG 1/2/3 in brackets.

During follow-up, there were no statistically significant differences between the occurrence of complex VPC between PH subgroups (detailed data not shown).

#### 3.2.3. Changes in heart rhythm during Holter electrocardiogram follow-up

##### 3.2.3.1. Atrial tachyarrhythmias

Eleven patients had an altered heart rhythm during Holter ECG follow-up. Patients with a change from SR to AFib were in PH groups 1, 2, 4, and 5. Three patients had an anamnesis of CAD (Table 5).

**Table 5:** Patients with changing heart rhythm during Holter electrocardiogram follow-up.

PH group	CAD	PVC burden			PAC	Heart Rhythm			BNP (pg/ml)		
		1	2	3		1	2	3	1	2	3
2	No	Lower	Lower	Lower	Yes	SR	SR	EAT	–	–	–
5	No	Lower	Lower	Higher	Yes	SR	AFib	AFib	442	1,121	400
1	Yes	Lower	Lower	Lower	No	SR	AFib	SR	120	333	80
4	No	Lower	Lower	Higher	Yes	SR	AFib	AFib	235	–	–
2	Yes	Lower	Lower	Higher	No	SR	AFib	SR	–	–	–
1	No	Lower	Lower	Lower	No	SR	SR	AFib	–	–	–
4	No	Higher	Higher	Higher	No	SR	AFib	SR	284	250	241
2	No	Lower	Higher	Higher	No	AFib	AFib	SR	–	–	–
2	No	Lower	Lower	Lower	Yes	AFib	SR	PMR	–	–	–
4	Yes	Lower	Lower	Higher	No	AFib	PMR	SR	–	–	–
5	No	Lower	Lower	Higher	Yes	AFib	AFib	SR	–	–	–

CAD, coronary artery disease; EAT, ectopic atrial tachycardia; AFib, atrial fibrillation; PMR, pacemaker rhythm; SR, sinus rhythm; PAC, premature atrial contractions, PVC, premature ventricular contractions, nsVT, non sustained ventricular tachycardia.

##### 3.2.3.2. Non-sustained ventricular tachycardias

Six patients had nsVTs during the first Holter ECG. Non sustained VT episodes occurred in 13 patients during the Holter ECG follow-up. In most patients, nsVT was found during follow-up. Patients with nsVT were from all PH groups (Table 6). In all patients, the first Holter ECG already revealed multiform/repetitive PVC. BNP values, sPAP and TAPSE showed no clear association to PVC burden or occurrence of nsVT (further information available in Supplement S1).

**Table 6:** Patients with non-sustained ventricular tachycardia (nsVT) during Holter electrocardiogram follow-up.

PH Group	CAD	PVC burden			PAC	Heart Rhythm		
		1	2	3		1	2	3
5	No	Lower	Lower	Higher	Yes	SR	AFib	AFib
3	Yes	Lower	Higher	Lower	No	SR	SR	SR
3	No	Lower	Lower	Higher	Yes	SR	SR	SR
4	No	Lower	Lower	Higher	Yes	SR	SR	SR
4	Yes	Lower	Lower	Higher	No	AFib	PMR	SR
4	Yes	Lower	Higher	Higher	Yes	SR	SR	SR
1	No	Lower	Higher	Higher	Yes	SR	SR	SR
4	No	Lower	Lower	Higher	Yes	SR	AFib	AFib
3	No	Lower	Higher	Lower	No	AFib	AFib	AFib
3	Yes	Lower	Lower	Higher	Yes	SR	SR	SR
2	Yes	Lower	Higher	Higher	No	SR	AFib	SR
2	No	Lower	Higher	Higher	No	AFib	AFib	SR
5	No	Lower	Lower	Higher	Yes	AFib	AFib	SR
4	No	Higher	Lower	Lower	No	–	–	SR
3	No	Higher	Lower	Lower	Yes	SR	SR	SR
4	No	Higher	Higher	Higher	No	SR	AFib	SR
1	No	Higher	Lower	Higher	Yes	SR	SR	SR
2	No	Higher	Lower	Lower	No	SR	SR	SR
1	No	Higher	Lower	Higher	No	SR	SR	–

BNP, brain natriuretic peptide; CAD, coronary artery disease; AFib, atrial fibrillation; PMR, pacemaker rhythm; SR, sinus rhythm; PAC, premature atrial contractions; PVC, premature ventricular contractions.

#### 3.2.4. Heart rhythm and survival

##### 3.2.4.1. Atrial fibrillation and survival

**Figure 4:**
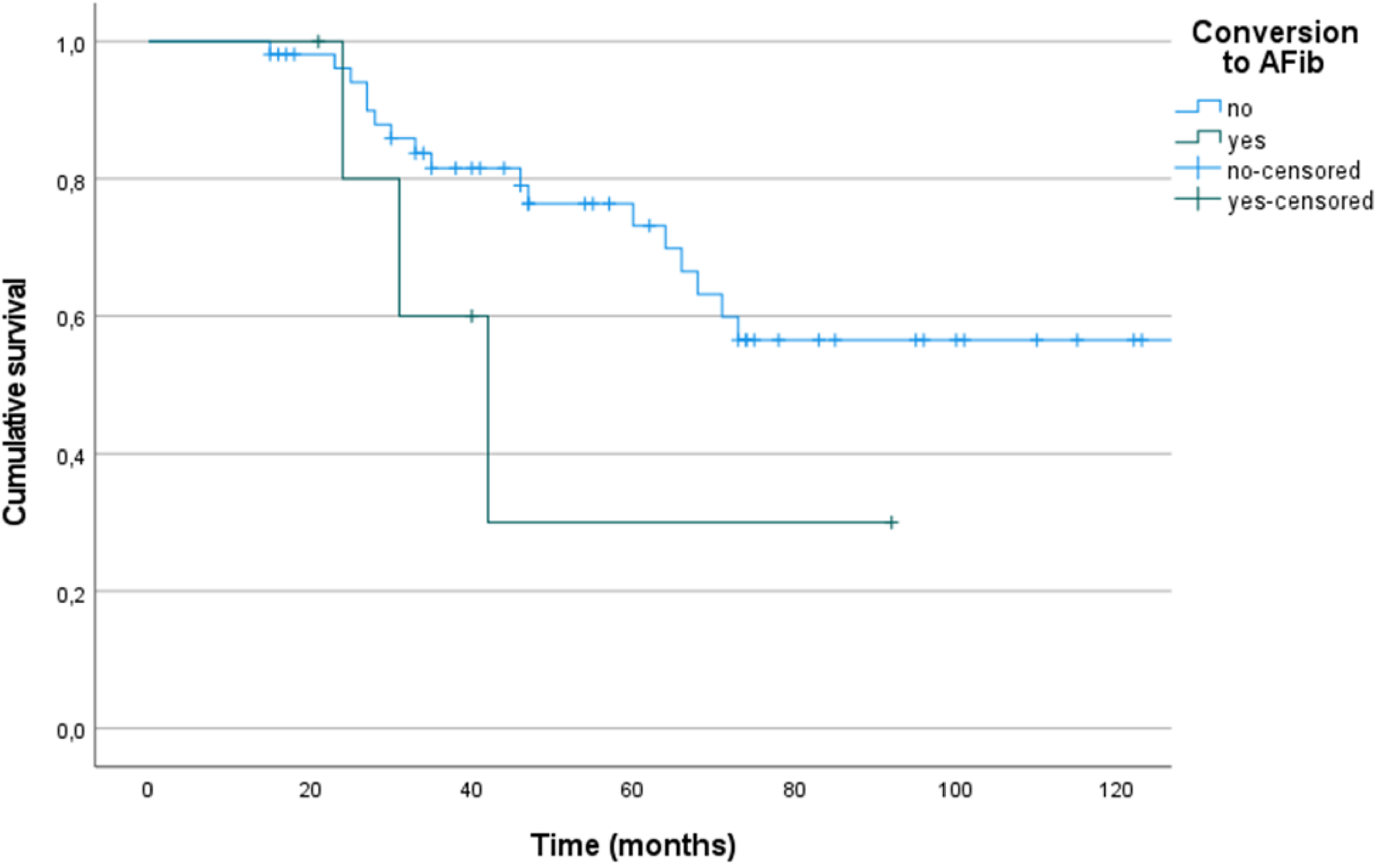
Kaplan–Meier plots (+ = censored data) for survival of patients with sinus rhythm (*n* = 48) and atrial fibrillation (AFib, *n* = 11).

Between patients (*n* = 11) with conversion to AFib during the three Holter ECGs no statistically significant difference of survival was found (1-, 3-, and 5-year survival: SR 100%, 79% and 73.2%, respectively, AFib 100%, 60% and 30%, respectively; log rank test *p* = 0.131; Figure 4).

##### 3.2.4.2. Non-sustained ventricular tachycardia and survival

**Figure 5:**
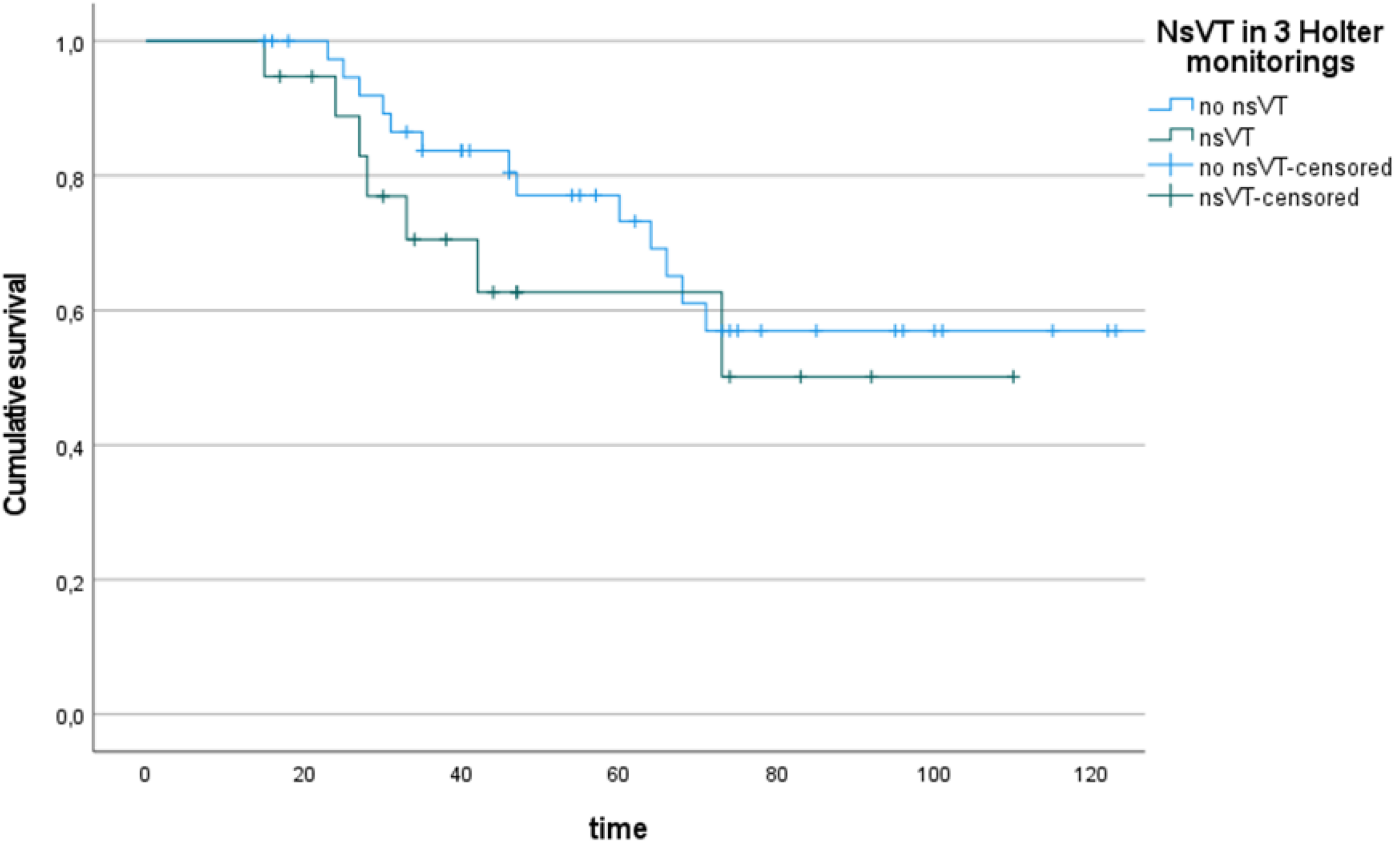
Kaplan–Meier plots (+ = censored data) for survival of patients with sinus rhythm (*n* = 40) and non-sustained ventricular tachycardia (nsVT, *n* = 19).

Analysis of survival revealed no statistically significant differences between patients with nsVT during Holter ECG monitoring and patients with permanent SR during follow-up (1-, 3-, and 5-year survival: SR 100%, 83.7% and 73.3%, respectively, nsVT 100%, 70.6% and 62.7%, respectively; log rank test *p* = 0.377; Figure 5).

## 4. Discussion

To our knowledge, our study is the first work to monitor patients with PH with repetitive Holter ECGs over a longer time [14 months (IQR 6–34)] and a long-term follow-up (120 months). The study contains two parts: First, to match our data with other studies (8, 10–12), we enrolled patients with group 1 and 4 PH (**part 1**). The first diagnosis of PH was made within the previous 12 months to minimise the potential influence of longstanding PH related effects on the heart leading to arrhythmias (30). Second, to follow up heart rhythm we analysed another subgroup including patients of all PH aetiologies with three follow-up Holter ECGs (**part 2**). We discuss the two parts of the study separately.

### 4.1. Part 1: Patients with PH groups 1 and 4

In contrast to other studies, only few patients (*n* = 4/65, 6.15%) had supraventricular tachycardia (AFib). In other studies, the incidence of supraventricular tachycardia has varied between 11.7% and 46.4% over a follow-up interval of 6–13 years (31). These results are in accordance with findings in two recent studies (12/43 and 162/641 patients) (32, 33). Interestingly, the number of patients with nsVT was high in our cohort [19/59 (32.2%); *n* = 6 during first Holter ECG, *n* = 13 during second/third Holter-ECG]. These findings are in contrast to most other studies with an incidence of nsVT between 5.9% and 13% (13, 32). Likewise, in other studies patients with nsVT mainly had group 2 PH (group 1 vs. 2, 15% vs. 48%) (34). A slightly higher prevalence than the aforementioned studies (but still clearly lower than in our study) was found recently with 16.7% of patients having nsVT in a cohort of patients with PH of all groups (group 1, 2.5%; group 3, 3.1%; group 4, 1.8%; group 5, 0%) (35).

Most of the studies evaluating survival revealed a statistically significant shorter survival of patients with supraventricular tachycardia (8, 12, 36–39). In contrast to these results, in two studies survival of patients with supraventricular tachycardia was not significantly shorter (15) or had no influence on survival (37). In our study, the small number of patients with supraventricular tachycardia (AFib, *n* = 4) did not allow any conclusion regarding survival time.

The incidence and prognostic value of PAC and PVC in patients with PH has not been investigated in studies before. The current AHA/ACC/HRS guidelines do not mention patients with PH (17).

Due to the individuality of quantification of PAC the results regarding the survival data of patients with PAC are difficult to assess. Our patients with PAC showed a trend towards shortened survival. In another study evaluating the prognostic relevance of PAC in patients with different indications for Holter ECG the burden of PAC was independently associated with mortality (40). In their meta-analysis, Himmelreich et al. (2019) found that frequent PAC during Holter ECG were associated with AFib (41). In contrast, our data showed PAC during Holter ECG in 4/11 patients with AFib for one or more of the three Holter ECGs.

Regarding survival of patients with nsVT, the data in the present study are consistent with our previous study (42).

### 4.2. Part 2: Patients of all PH aetiologies with three follow-up Holter ECGs

SR was the most common rhythm in our patients. LVEF (during Holter ECG 1) was normal in nearly all patients. CAD was present in 16/59 patients (PH group 1: 5/14; group 2: 4/9; group 3: 3/12; group 4: 4/19; group 5: 0/2), especially in 3/11 patients with AFib and in 5/19 patients with nsVT. These findings indicate that in patients with PH, mechanisms other than ischaemia/CAD seem to be responsible for arrhythmias (see below).

Holter ECG monitoring revealed PAC frequently, but all in all, the distribution was without statistically significant differences between PH groups. PVC occurred often with high frequency in all PH groups, too. Witte et al. (2016) evaluated patients with PH who underwent 24-hour Holter ECG (43): Patients with PH group 1 had a higher burden of PVC and a decrease in HRV (SDNN). In patients with PH group 4, the number of PVC differed from controls (“higher total count of PVC”), whereas in patients with PH and COPD, both PAC and PVC were more frequent than in controls and SDNN was similar to patients with PH group 1. Another study also revealed frequent PVC and reduced HRV (SDNN) in PH patients compared with controls (44). Increased PAC, PVC, and arrhythmias are known to occur in patients with COPD (45, 46).

At the time of Holter ECG 1, patients (with three Holter ECGs) with nsVTs had higher values of sPAP and BNP. This association was less clear at the time of Holter ECGs 2 and 3. Therefore, sPAP and BNP values are not sufficient to identify patients with more frequent arrhythmias (or at risk for arrhythmias).

Another interesting observation is that patients with an initial occurrence of multiform/repetitive PVC at Holter ECG 1 continued to show complex PVC during follow-up. All patients who showed conversion to AFib had multiform/repetitive PVC. These findings may indicate greater impairment of right heart function.

In patients converting to AFib and patients with prevalent nsVT no statistical significant differences in survival was found. It must be noted that in our study the number of patients developing supraventricular arrhythmia (AFib) was small (*n* = 11). Most of the studies evaluating survival have revealed significantly shortened survival of patients with supraventricular tachycardia (8, 12, 36–39). However, some studies have revealed no significant difference (15, 39).

Based on the literature, different causes seem to be responsible for arrhythmias in patients with PH. Umar et al. (2012) found that in rats with PH induced by subcutaneous injection of monocrotaline, early afterdepolarisations (EADs) from the right ventricular epicardial surface triggered ventricular tachycardia. The study revealed two underlying mechanisms that maintain ventricular arrhythmias: focal and incomplete re-entrant wave fronts during ventricular fibrillation (6).

Rosas-Peralta et al. (2006) revealed an autonomic cardiac disturbance in patients with group 1 and 2 PH (Eisenmenger syndrome). Increased sympathetic tone led to loss of the circadian rhythm of HRV. After administering treprostinil, there was a recovery of sympathovagal balance. The authors concluded the changes may be markers of autonomic dysbalance that favour the development of arrhythmias and SCD. The sympathovagal balance in PH could be considered an important risk marker (47).

Another important cause for arrhythmias in patients is cardiac fibrosis. In rats with PH, increased myocardial fibrosis generates the substrate for the initiation and maintenance of ventricular arrhythmias (6, 48, 49). Cardiomyocytes from a failing right ventricle exhibited prolonged action potential duration, EADs, triggered activity, and spontaneous re-entrant ventricular arrhythmias caused by right-sided AP alternans at physiological rates (50).

Noteworthy, in a basic science study with rats receiving monocrotaline leading to advanced PAH, the treated rats showed prolongation of action potential duration (APD), increased APD heterogeneity and other changes, which led to rapid pacing and susceptibility to sustained VT.

A selective intra-tracheal gene delivery (of aerosolized AAV1 carrying S2a resulting in selective upregulation of the human isoform of SERCA2a) in the lung but not the heart improved monocrotaline-induced prolongation of cardial action potential duration and thus suppressed the incidence of pacing-induced VT (51).

Histology studies in humans have revealed right ventricular fibrosis in patients with PAH (52, 53). These results are consistent with electrophysiological findings in patients with PH, which have revealed cardiac fibrosis. However, the experience with electrophysiology studies in patients with PH is very limited. Medi et al. reported that patients with right atrial dilatation/remodelling showed a reduction in conduction velocity and an increase in areas of low voltage or electrical silence due to interstitial fibrosis (54).

In a patient with PH caused by atrial septal defect and partial anomalous pulmonary venous drainage, electrophysiology studies revealed a non-sustained focal activation and its origin very close to a small low-voltage area at the lateral wall of the right ventricle near the tricuspid annulus, suggesting structural remodelling of the RV (55).

Another invasive tool used to estimate cardiac fibrosis is electroanatomic mapping (high-density peak-to-peak voltage mapping). A taxable and locatable electrophysiological catheter is introduced into the right ventricle using electro-anatomical and additive fluoroscopic guidance to receive three-dimensional geometry and a peak-to-peak voltage and scar map. In patients with arrhythmogenic right ventricular dysplasia, researchers noted concordance between electroanatomic findings and magnetic resonance imaging (MRI) or echocardiographic findings (56).

A new non-invasive tool for the diagnosis of cardiac fibrosis is MRI. Myocardial tissue characterisation in PAH is feasible using late gadolinium enhancement and T1 values (57). In the future, it will be interesting to use MRI to quantify the fibrosis degree in patients with ventricular arrhythmia to determine whether the fibrosis degree correlates with the grade of ventricular arrhythmic burden and risk of arrhythmias or SCD in general.

### 4.3. Limitations

Our study is not free of limitations: It was conducted as a single center study. Because PH is a rare disease, only a relatively small number of patients could be included. The retrospective design of the study (missing data) and the number of different examiners meant that no standardised findings of the Holter ECGs were available. The frequency of PAC during Holter ECG was stated subjectively by the physician (qualitative/quantitative). A more extensive rhythm monitoring was carried out by Holter ECG monitoring, but it must be noted that this is not a full rhythm monitoring over a longer period of time.

Finally, based on the study design the follow up scheme was set due to clinical need and not specific study requirements, which has impact on survival analyses and the follow-up Holter monitorings. However, especially the interval between the different Holter monitoring dates is considered acceptable.

## 5. Conclusion

Our present study was conducted to detect the arrhythmic burden in PH and its potential prognostic significance. In our PH cohort patients with PAC tend to a shortened survival, in contrast the occurrence of nsVT was not linked to an impaired survival prognosis. None of the evaluated parameters (BNP, TAPSE, sPAP) was correlated with the development of arrhythmias. Patients showing multiform/repetitive PVC seem to stay at risk for complex ventricular arrhythmias over a longer time in the disease process. Further research to verify our results with the inclusion of a higher number of subjects, preferably within the frame of multicentric studies, is desirable.

## Data Availability

The raw data supporting the conclusions of this article will be made available by the authors, without undue reservation.
