# Supplementary material for: Incidence and prognostic significance of malignant arrhythmias during (repetitive) Holter electrocardiograms in patients with pulmonary hypertension
Source: Front Cardiovasc Med. 2023 Apr 17;10:1084051. doi: 10.3389/fcvm.2023.1084051 (PMC10150006; doi:10.3389/fcvm.2023.1084051)
Supplement: Supplementary file 1 [file Datasheet1.docx]

**Supplement 1**

Premature atrial contractions, premature ventricular contractions burden and brain natriuretic peptide, systolic pulmonary arterial pressure and tricuspid annular plane systolic excursion during Holter ECG Follow-Up.

| PH group | CAD | PVC burden | | | PAC | Heart rhythm | | | BNP  (pg/ml) | | | sPAP  (mmHg) | | | TAPSE  (mm) | | |
| --- | --- | --- | --- | --- | --- | --- | --- | --- | --- | --- | --- | --- | --- | --- | --- | --- | --- |
|  |  | 1 | 2 | 3 |  | Holter ECG 1 | Holter ECG 2 | Holter  ECG 3 | 1 | 2 | 3 | 1 | 2 | 3 | 1 | 2 | 3 |
| 5 | No | Lower | Lower | Higher | Yes | SR | AFib | AFib | 442 | 1121 | 400 | 65 | 68 | 67 | 16 | 12 | 15 |
| 3 | Yes | Lower | Higher | Lower | No | SR | SR | SR | 8 | 14 | - | 30 | 36 | 24 | - | 26 | - |
| 3 | No | Lower | Lower | Higher | Yes | SR | SR | SR | 63 | 60 | 342 | 45 | 55 | 52 | 18 | 18 | 18 |
| 4 | No | Lower | Lower | Higher | Yes | SR | SR | SR | 64 | - | - | 67 | 63 | 61 | 21 | 25 | 21 |
| 4 | Yes | Lower | Lower | Higher | No | AFib | PMR | SR | 96 | 152 | - | 98 | 54 | 60 | - | - | 13 |
| 4 | Yes | Lower | Higher | Higher | Yes | SR | SR | SR | - | - | - | 44 | 42 | 53 | - | 21 | 16 |
| 1 | No | Lower | Higher | Higher | Yes | SR | SR | SR | 706 | 765 | 445 | 80 | 80 | 78 | - | - | 13 |
| 4 | No | Lower | Lower | Higher | Yes | SR | AFib | AFib | 235 | - | - | 53 | 51 | - | 23 | 18 | 14 |
| 3 | No | Lower | Higher | Lower | No | AFib | AFib | AFib | 191 | - | - | 56 | 19 | 53 | 15 | 19 | 17 |
| 3 | Yes | Lower | Lower | Higher | Yes | SR | SR | SR | 64 | 48 | - | 51 | - | 47 | - | - | - |
| 2 | Yes | Lower | Higher | Higher | No | SR | AFib | SR | - | - | - | 50 | 33 | 33 | - | - | - |
| 2 | No | Lower | Higher | Higher | No | AFib | AFib | SR | - | - | - | 50 | 50 | 46 | - | - | - |
| 5 | No | Lower | Lower | Higher | Yes | AFib | AFib | SR | 289 | 462 | 606 | 42 | 65 | 71 | 28 | 21 | 18 |
|  | | | | | | | | | | | | | | | | | |
| 4 | No | Higher | Lower | Lower | No | - | - | SR | - | - | - | 45 | 45 | 53 | - | - | - |
| 3 | No | Higher | Lower | Lower | Yes | SR | SR | SR | 795 | 794 | 314 | 77 | 77 | 59 | - | - | - |
| 4 | No | Higher | Higher | Higher | No | SR | AFib | SR | 284 | 250 | 241 | 65 | 78 | 61 | - | - | - |
| 1 | No | Higher | Lower | Higher | Yes | SR | SR | SR | 108 | 383 | 273 | 92 | 85 | 105 | 31 | - | - |
| 2 | No | Higher | Lower | Lower | No | SR | SR | SR | - | - | - | - | - | - | 17 | 15 | 12 |
| 1 | No | Higher | Lower | Higher | No | SR | SR | - | 123 | - | - | 74 | 42 | 60 | 21 | 19 | 15 |

BNP, brain natriuretic peptide; CAD, coronary artery disease; AFib, atrial fibrillation; SR, sinus rhythm; PAC, premature atrial contractions; PVC, premature ventricular contractions; sPAP, systolic pulmonary arterial pressure; TAPSE, tricuspid annular plane systolic excursion; PMR, pacemaker rhythm.
